# Efficacy of a Modified Superficial Temporal Artery–Middle Cerebral Artery Bypass Using Superficial Temporal Artery Side-Branch Donors in Adult Moyamoya Disease: A Technical Note

**DOI:** 10.3390/jcm14196904

**Published:** 2025-09-29

**Authors:** Shintaro Arai, Tatsuya Sugiyama, Tohru Mizutani, Kenji Sumi, Masaki Matsumoto, Kouzou Murakami, Ryo Irie, Yoichi Morofuji

**Affiliations:** 1 Department of Neurosurgery, School of Medicine, Showa Medical University, 1-5-8 Hatanodai, Shinagawa-ku 142-8555, Tokyo, Japan; sumiken202@med.showa-u.ac.jp (K.S.); me13078@med.showa-u.ac.jp (M.M.); morofujiyoichi@gmail.com (Y.M.); 2 Department of Neurosurgery, Koto-Toyosu Hospital, Showa Medical University, 5-1-38 Toyosu, Koto-ku 135-8577, Tokyo, Japan; tasugiyama@med.showa-u.ac.jp (T.S.); mizutani.nsutky@gmail.com (T.M.); 3 Department of Radiology, School of Medicine, Showa Medical University, 1-5-8 Hatanodai, Shinagawa-ku 142-8555, Tokyo, Japan; kouzou013@gmail.com; 4 Department of Neurosurgery, Fujigaoka Hospital, Showa Medical University, 1-30 Fujigaoka, Aoba-ku, Yokohama 227-8501, Kanagawa, Japan; sinus.iridum0131@gmail.com

**Keywords:** moyamoya disease, direct bypass, superficial temporal artery, side-branch donor, hyperperfusion, revascularization

## Abstract

**Background:** Adult moyamoya disease (MMD) is a progressive steno-occlusive cerebrovascular disorder for which surgical revascularization is the primary treatment. The standard direct superficial temporal artery–middle cerebral artery (STA-MCA) bypass uses the frontal and/or parietal branch of the STA as the donor. However, in some patients, conventional STA-MCA bypass may be suboptimal because of a large mismatch in caliber between the STA branch and the recipient artery, increasing the risk of cerebral hyperperfusion. This study aimed to investigate the impact of a modified STA-MCA bypass on MMD treatment. **Methods:** We retrospectively reviewed adult cases of MMD at our institution (2012–2025) for patients who underwent modified direct STA-MCA bypass using a small side branch of the STA as the donor artery. Surgical techniques and clinical outcomes of these cases were analyzed descriptively. **Results:** Five cases (five hemispheres in four patients) underwent side-branch STA-MCA bypass. All procedures were completed successfully, with 100% graft patency confirmed by intraoperative indocyanine green angiography, and a mild increase in cerebral blood flow confirmed by postoperative single-photon emission computed tomography. No patients developed postoperative cerebral hyperperfusion syndrome or wound healing complications. Clinically, all patients experienced a stable or improved neurological status, with no reported new ischemic or hemorrhagic events during follow-up. **Conclusions:** In this small feasibility series, the side-branch STA–MCA bypass was technically feasible and safe, with no cerebral hyperperfusion syndrome observed. Any risk-mitigating effect on hyperperfusion remains theoretical and requires confirmation in comparative studies.

## 1. Introduction

Moyamoya disease (MMD) is an idiopathic chronic steno-occlusive cerebrovascular disorder characterized by progressive narrowing of the intracranial internal carotid arteries and their main branches, with development of abnormal basal collateral vessels (“moyamoya vessels”). The primary treatment goals for MMD are to increase cerebral blood flow (CBF), improve cerebrovascular reserve, and prevent future ischemic or hemorrhagic stroke through revascularization. Surgical revascularization, either direct, indirect, or a combination of both, is the mainstay of therapy for achieving these aims [1,2]. In particular, direct superficial temporal artery–middle cerebral artery (STA-MCA) bypass has become the gold standard for adult ischemic MMD as it provides immediate augmentation of CBF.

**Figure 1:**
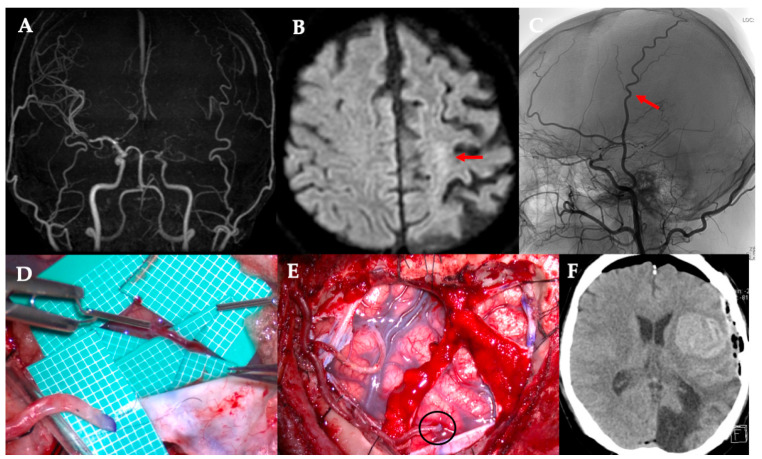
Demonstration of a 46-year-old man underwent left-sided revascularization for Moyamoya disease with an ischemic onset. A difference in the caliber was observed between the donor and recipient arteries. The patient was postoperatively managed with sedative intubation due to concerns regarding the risk of hyperperfusion. Immediately after surgery, CT showed extensive cerebral hemorrhage. (**A**) MRA. Poor delineation of bilateral internal carotid artery endings was observed. (**B**) DWI. Left acute cerebral infarction was observed (red arrow). (**C**) STA parietal branch was particularly well developed (red arrow). (**D**,**E**) The STA parietal branch trunk (2 mm) was anastomosed to M4 of the frontal lobe (0.9 mm, black circle). (**F**) Immediately after surgery, extensive cerebral hemorrhage is observed in the left frontal lobe. CT, computed tomography; DWI, diffusion-weighted image; MRA, magnetic resonance angiography; STA, superficial temporal artery.

Despite its effectiveness, direct bypass surgery in adult patients with MMD remains challenging. One major concern is that STA donors may not always be optimally suited for anastomosis. The “orthodox” STA-MCA bypass uses one STA branch (frontal or parietal) for a single bypass, or both branches for a double-barrel bypass if broader coverage is needed. However, in some cases, the available cortical recipient artery is very small (diameter < 1 mm), and a larger donor artery can produce a flow surplus and technical difficulty in suturing because of a caliber mismatch. Furthermore, bypass of small recipient vessels is associated with an increased risk of hyperperfusion [3]. Our institutional experience includes cases of cerebral hemorrhage resulting from hyperperfusion following bypass with a significant donor–recipient caliber mismatch (Figure 1). In such situations, conventional bypass using the main STA branch may not be ideal.

Various strategies have been explored to address these issues. If an STA is unavailable or unsuitable, alternative donor arteries, such as the occipital artery and the posterior auricular artery (PAA), can be employed. For example, Torazawa et al. reported a successful long-term PAA-MCA bypass in an adult patient with MMD as a rescue procedure when the STA was not available [4]. Other innovations include the modification of the bypass technique. For instance, Lu et al. recently reported a modified “dual-branch” STA-MCA bypass technique in which both STA branches are utilized (one for direct anastomosis and one for indirect “patch” synangiosis), resulting in lower hyperperfusion rates and improved collateralization compared to conventional surgery [5]. In a separate approach, Lawton et al. described a side-to-side STA-MCA bypass that preserved the continuity of the STA, thereby maintaining the flow to native collaterals while forming a direct anastomosis [6]. These modifications have shown promise in reducing complications and improving outcomes in adult patients with MMD; however, further studies are needed.

This study aimed to describe the effect of a modified STA-MCA bypass using only an STA side branch as the donor used in our institution and the initial clinical results in a series of adult patients. The findings of this study could aid in the treatment of MMD.

## 2. Materials and Methods

### 2.1. Patient Selection

We retrospectively evaluated 50 consecutive cases of MMD treated by a single surgeon between 2012 and 2025. All patients underwent combined direct and indirect revascularization. From this cohort, we identified patients who underwent a modified direct STA-MCA bypass using an STA side-branch donor. Five cases (five hemispheres in four patients) were included. Patients (aged >18 years) had angiographically confirmed MMD and presented with either ischemic symptoms or hemorrhagic stroke. The modified bypass was selected intraoperatively when the operating surgeon judged that conventional STA-MCA anastomosis using the main STA branch was not ideal (for example, due to a very small recipient artery or suboptimal STA anatomy). Clinical data and operative records were reviewed for all the included cases.

This series comprised five hemispheres in four adult patients. Unless otherwise specified, operative and imaging outcomes are summarized per hemisphere (*n* = 5); clinical outcomes including modified Rankin Scale (mRS) are summarized per patient (*n* = 4).

This study was approved by our institutional review board and conducted in accordance with the ethical standards of the 1964 Declaration of Helsinki and its later amendments. Instead of obtaining written informed consent from all participants, an opt-out notice was posted on our institutional website prior to patient inclusion in this study.

### 2.2. Surgical Technique

All surgeries were performed under general anesthesia, with the patient’s head fixed in the supine position. A curvilinear frontotemporal skin incision was made to expose the STA and its branches. Preoperative computed tomography angiography (CTA), magnetic resonance angiography (MRA), or digital subtraction angiography (DSA) were used to evaluate STA anatomy and identify suitable M4 cortical recipients. Under an operating microscope, the STA—including its trunk and parietal (temporal) branch—was dissected free from the surrounding scalp tissue and preserved in all cases. The frontal branch was dissected and preserved only in cases where bypass was performed at two sites, allowing donor options to be optimized accordingly.

After exposing the recipient artery, the final donor selection (trunk vs. side branch) was based on direct in vivo assessment of vessel caliber and reach without tension. Calibers of the donor side branch and M4 recipient vessels were measured in vivo intraoperatively using a rubber sheet with micro-measurements. Each measurement was performed by a single observer, and values were confirmed by an independent second observer to ensure reproducibility.

In the modified approach, a small-caliber STA side branch—originating from either the frontal or parietal division—was chosen as the donor vessel. It was mobilized sufficiently to reach the recipient site while maintaining its proximal connection to the STA trunk. The STA trunk and other branches were left intact to maintain scalp perfusion.

A standard frontotemporal craniotomy or craniectomy was performed. The dura was opened, preserving meningeal STA branches when present. The M4 cortical branch of the MCA, typically within the ischemic region, was selected as the recipient. After temporary microclip placement, an arteriotomy was performed, and an end-to-side anastomosis between the STA side branch and MCA branch was completed using interrupted 10-0 nylon sutures. Temporary clips were then removed to restore flow.

Bypass patency was confirmed intraoperatively with indocyanine green (ICG) videoangiography in all cases. No revisions were needed. After hemostasis, the dura was loosely closed. The STA and its remaining branches were laid on the brain surface as an indirect bypass. The bone flap was replaced without graft compression, followed by standard closure of the galea and skin.

### 2.3. Postoperative Management and Follow-Up

All patients were administered a single antiplatelet drug in the preoperative period to promote graft patency and were monitored in an intensive care unit with strict blood pressure control to mitigate the risk of hyperperfusion. Neurological examinations were frequently performed. Signs of cerebral hyperperfusion syndrome (CHS; e.g., headache, seizures, and focal deficits) were specifically observed during the first week after surgery. Single-photon emission computed tomography (SPECT) was performed within 1–2 days after surgery to evaluate hemodynamics. The ratio of the resting blood flow in the bypassed region of interest (A: mL/min/100 g) to that in the contralateral region of interest (B: mL/min/100 g) was calculated (A/B), and the rates of increase in this ratio before and after surgery were measured. Hospital discharge occurred approximately 2 weeks after surgery in each case. Follow-ups included outpatient clinical visits and brain imaging. Follow-up imaging was scheduled with DSA at 1 year in principle. In addition, all patients underwent serial magnetic resonance imaging (MRI) examinations during the follow-up period. Wound events were monitored before discharge and at each subsequent outpatient visit. Scalp trophism was evaluated by visual inspection and palpation.

The clinical outcome measures at the last follow-up included any recurrent ischemic events (transient ischemic attacks [TIAs] or strokes), hemorrhagic events, and the patient’s functional status (mRS). No formal statistical analysis was performed because of the small sample size of this case series. The results were presented descriptively.

## 3. Results

### 3.1. Patient Characteristics and Surgical Details

Five cases (five hemispheres in four patients; age range, 36–55 years; mean age, 45 years) underwent modified STA side-branch bypass during the study period. Three patients presented with ischemic symptoms, and one patient presented with an intracerebral hemorrhage. Angiographically, the operated hemisphere showed Suzuki stage III–V disease. In all cases, the side branch of the STA parietal division was used as the donor, because the branch had a suitable small-caliber offshoot and an adequate length to reach a cortical recipient.

**Figure 2:**
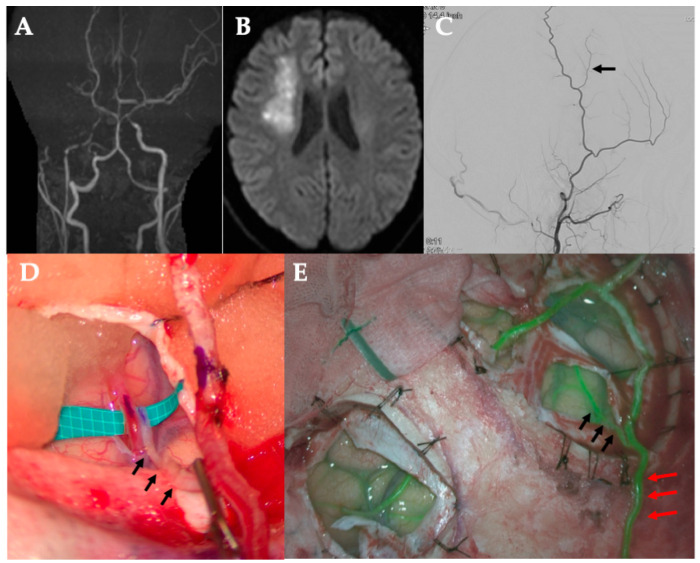
Case 1. (**A**) MRA. Poor delineation of bilateral internal carotid artery endings. (**B**) DWI. Right acute cerebral infarction. (**C**) Revascularization using the side of the STA parietal branch (black arrow). (**D**,**E**) The STA parietal branch main stem blood flow is preserved (red arrow), and the side branch (black arrow) is anastomosed to M4 in the frontal lobe. ICG showed patency of the bypass vessel (black arrow). MRA, magnetic resonance angiography; DWI, diffusion-weighted image; STA, superficial temporal artery; ICG, indocyanine green.

**Figure 3:**
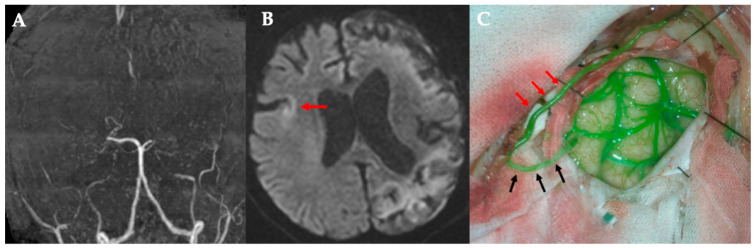
Case 2. The case of rapidly progressive MMD. (**A**) MRA. The main trunk of the internal carotid artery system has almost disappeared. (**B**) DWI. Right acute cerebral infarction occurred (red arrow) after the bypass surgery for the left cerebral hemisphere. (**C**) The side branch of the right STA parietal branch (black arrow) is anastomosed to M4 of the frontal lobe (black arrowhead), while blood flow is preserved in the main trunk (red arrow). MMD, moyamoya disease; MRA, magnetic resonance angiography; DWI, diffusion-weighted image; STA, superficial temporal artery.

**Figure 4:**
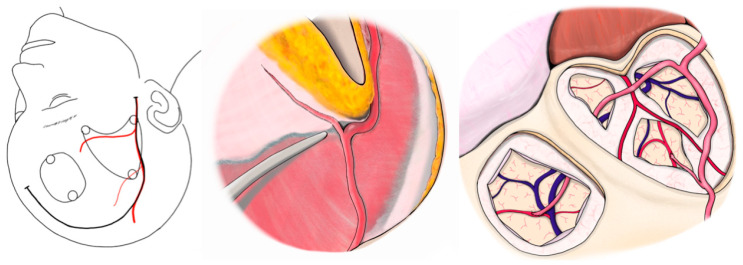
Case 1. Scheme of modified STA-MCA bypass using STA side-branch donors. STA, superficial temporal artery; MCA, middle cerebral artery.

All five procedures were technically successful. Table 1 summarizes the key characteristics of each case. Preserving a side branch added an average of 5.6 min to the procedure (range, 3–11 min). In all cases, the bypass was patent on intraoperative ICG angiography, which demonstrated immediate perfusion of the distal MCA territory through the graft (Figure 2 and Figure 3). A schematic of this procedure is shown in Figure 4. The caliber of the STA side-branch donors ranged from approximately 0.5 mm to 1.1 mm (mean: 0.88 mm), closely matching the recipient artery diameters (0.4–0.9 mm, mean: 0.64 mm). No intraoperative complications (such as anastomotic thrombosis and vessel injury) occurred. The main STA trunk and any unused branches remained in situ to continue perfusing the scalp, and no signs of scalp ischemia were observed after sacrificing the side branch.

### 3.2. Postoperative Outcomes

None of the patients in this series developed CHS after surgery. There were no instances of transient neurological deterioration attributable to hyperperfusion, and none of the patients experienced seizures or intracerebral hemorrhage in the postoperative period. The mean increase in CBF before and after surgery on SPECT was 1.17 (1.04–1.34). All patients recovered without complications. The scalp incisions healed well in all cases, and no wound complications (such as scalp necrosis or infection) were observed. Notably, we did not encounter any issues with scalp flap perfusion, probably because the main STA was preserved in all cases to continue providing blood supply to the scalp.

During a mean follow-up of 17.4 months (range, 5–36 months), none of the four patients experienced any new ischemic stroke or hemorrhagic events. The baseline mRS was median 3 (interquartile range [IQR] 2–5) and improved to 0.5 (IQR 0–3) at last follow-up (*n* = 4). Four patients with preoperative ischemic symptoms reported improvement or resolution of symptoms after bypass. Specifically, patients with TIAs had no recurrence of TIA after surgery, and patients with a prior minor infarction showed improved motor function in the affected extremity. The patient who presented with an intracerebral hemorrhage did not experience any episodes of re-bleeding after revascularization. In practice, none of the patients underwent DSA at 1 year. Four cases were followed with angiography at different timepoints: postoperative day 7, 2 months, and 6 months for Case 1; postoperative day 7, 6 months, and 9 months for Case 3; 1 month postoperatively for Case 4; and postoperative day 7 for Case 5. All patients were also followed with serial MRI examinations at varying intervals (Table 2). Follow-up angiography (MRA or catheter angiography) revealed a patent bypass graft in all cases. In each patient, the revascularized hemisphere demonstrated improved perfusion and collateral circulation compared to the preoperative studies.

## 4. Discussion

In this case series, we demonstrated that a modified STA-MCA bypass using only an STA side branch as the donor is a feasible and effective surgical option for adult patients with MMD in selected scenarios. All five cases achieved successful revascularization with this technique, as evidenced by 100% bypass patency and the absence of postoperative CHS or other major complications. These initial results suggest that using a smaller-caliber STA branch can provide sufficient cerebral perfusion while potentially mitigating the risks associated with a standard high-flow STA graft in certain patients.

The avoidance of CHS in our study was a notable finding. CHS is a well-recognized complication of direct bypass surgery for MMD, especially in adults with impaired autoregulation. Uchino et al. reported that symptomatic hyperperfusion occurred in 31.5% of adult patients with MMD after direct bypass, compared to only 5% in children [7]. The risk of CHS correlates with excessive flow through the bypass relative to the capacity of the recipient bed. Using a small side-branch donor, our technique inherently limits the flow introduced into the intracranial circulation. The narrower lumen and lower flow capacity of the side branch act as a natural “flow optimizing,” potentially preventing the sudden overperfusion of the microvasculature that can occur with a larger STA graft. Consistent with this observation, none of our five cases exhibited CHS, although the sample size was too small to draw definitive conclusions. Similarly, Lu et al. found that the modified dual-branch bypass approach was associated with a lower incidence of hyperperfusion (18.2% vs. 23.3%) and improved angiographic collateral grades compared to conventional bypass in adult patients with MMD [5]. Therefore, these findings support the concept that tailoring bypass flow to patient needs can improve safety.

Another advantage of the side-branch bypass technique is preservation of the scalp blood supply. Because we harvested only a small branch of the STA and left the main STA trunk intact, the overall perfusion of the scalp was less compromised than in a traditional double-barrel bypass (where both STA branches are used). Scalp wound healing complications have been associated with double-barrel bypass procedures that sacrifice both STA branches due to the extensive disruption of scalp circulation. No wound complications were observed in this study. All incisions healed primarily without issues, which is an encouraging outcome, particularly in patients with risk factors for poor wound healing (such as older age and comorbidities). By leaving the main STA in situ, our technique likely helps maintain adequate blood flow to the skin and subcutaneous tissues.

The concept of modifying bypass techniques to optimize outcomes has been gaining attention in the treatment of MMD. Traditionally, a single high-flow STA-MCA bypass has been the standard; however, there is increasing recognition that a more nuanced approach may benefit certain adult patients. Lawton et al. pioneered a side-to-side STA-MCA bypass to preserve native collateral flow and demonstrated that it was technically feasible and provided effective flow augmentation without sacrificing the continuity of the donor STA’s continuity [6]. Our approach differs in that we still perform an end-to-side anastomosis (sacrificing the side branch’s distal continuity), but we achieve flow moderation by selecting a smaller donor vessel. Both approaches aim to protect the brain from hemodynamic extremes, either by preserving collaterals or reducing flow, and neither approach was associated with CHS in the reported cases.

The efficacy of this side-branch bypass is supported by favorable clinical outcomes. All patients remained stroke-free during follow-up, and their preoperative ischemic symptoms improved or resolved. This suggests that although the bypass flow may be lower than that of a larger-caliber graft, it is sufficient to augment perfusion in chronic ischemic areas. It is well known that even modest increases in CBF can significantly reduce the risk of ischemic events in patients with MMD by improving hemodynamic reserve. Additionally, indirect collateral formation likely continued postoperatively in our patients (typically after revascularization), further augmenting CBF over time. We did not perform quantitative CBF measurements postoperatively in this small series. However, clinically, none of the patients showed signs of inadequate perfusion.

From a technical standpoint, the use of a small side branch as a donor requires careful microsurgery. Anastomosis is performed between two small vessels, which can be more technically demanding than the standard STA-MCA bypass. However, with modern microsurgical instruments and expertise, fine vessel anastomoses are now routinely performed by experienced cerebrovascular surgeons. In our study, all anastomoses were successfully completed without revision or intraoperative bypass failure. We found that ensuring a tension-free graft was critical for adequate mobilization of the side branch, and, if necessary, gentle dissection of the surrounding tissue was performed to maximize the reach. An interposition graft was not required in any case, and the inherent length of the STA side branches was sufficient for a short distance from the cortical surface.

It is important to acknowledge that the side-branch bypass technique is not intended to replace the standard STA-MCA bypass in all cases but rather to serve as an additional option tailored to specific circumstances. In cases in which the STA is large and the recipient artery is of good caliber, conventional direct bypass remains an excellent procedure with proven long-term efficacy. Conversely, in cases where donor–recipient mismatch or hyperperfusion risk is a major concern, our results indicate that opting for a smaller donor can be a prudent strategy. Thus, surgeons should consider the full spectrum of bypass techniques (single, double, side-to-side, and side-branch) and select the one that best fits the patient’s angiographic anatomy and hemodynamic profile.

Our study has some limitations. First, the number of cases was small (*n* = 5), limiting the generalizability of the findings. The absence of complications in five cases, although reassuring, could be due to chance, and larger studies are needed to determine complication rates more accurately. Second, this study was retrospective, without a control group. We did not directly compare the outcomes of our side-branch bypass with those of the conventional bypass in similar patients. Thus, we cannot conclusively claim that the modified technique is superior; only that it was successful and safe in this series. Third, we relied on clinical follow-up and non-invasive imaging to infer bypass patency and efficacy; formal angiographic confirmation of long-term patency was not obtained in every case. A future study with routine postoperative angiography at 6–12 months would be valuable for quantifying the extent of revascularization. The Matsushima grading system (Grade A, B, C) is commonly used to categorize the extent of collateral filling after indirect bypass and could be applied to assess our direct/indirect combined results as well [8]. We anticipate that our bypasses will achieve good collateral grades; however, we defer formal grading until angiographic data become available. Finally, long-term follow-up beyond 3 years is needed to ensure that the benefits of side-branch bypass persist and that no late complications occur.

Therefore, our initial experience suggests that the STA side-branch to MCA bypass is a viable adjunct in the surgical management of adult MMD. This technique provided effective revascularization in five cases who may have been at an elevated risk for hyperperfusion or technical issues with a standard bypass. This modification leverages the fundamental principle of microsurgery, matching the size of the donor and recipient vessels to improve the balance of flow. Although further evaluation in larger cohorts is warranted, STA side-branch bypass offers a promising option for surgeons to add to their armamentarium and tailor revascularization strategies to individual patient needs.

Future studies could incorporate direct intraoperative flow quantification to more precisely evaluate the hemodynamic impact of the side branch STA–MCA bypass. Such measurements would allow a more detailed comparison between the conventional STA–MCA bypass and the modified approach described in this study, potentially clarifying the theoretical benefits in hyperperfusion risk mitigation.

## 5. Conclusions

In this small feasibility series, the side-branch STA–MCA bypass was technically feasible and safe, with no CHS observed. Any risk-mitigating effect on hyperperfusion remains theoretical and requires confirmation in comparative studies.

## Figures and Tables

**Table 1:** Patient characteristics and results.

Case	Age (Years)	M4 Diameter (mm)	STA Side Branch Diameter (mm)	Increased Rate in CBF	Postoperative Complication	Duration of Follow-Up (Months)
1	47	0.6	1	1.28	no	36
2	55	0.9	1	1.11	no	26
3	36	0.4	0.8	1.08	no	14
4	36	0.5	0.5	1.04	no	6
5	43	0.8	1.1	1.34	no	5

CBF, cerebral blood flow; STA, superficial temporal artery.

**Table 2:** Follow-up imaging modality and timing for each patient.

Case	Angiography	MRI (Months Postop)
1	7 days, 2 months, 6 months	1, 6, 8, 10, 12, 18, 20, 22, 24, 36
2	-	1, 23, 26
3	7 days, 6 months, 9 months	2, 4, 8, 9, 10, 14
4	1 month	1, 2, 6
5	7 days	2, 3, 5

MRI, magnetic resonance imaging.

## Data Availability

The data presented in this study are available on request from the corresponding author due to institutional and ethical restrictions related to patient confidentiality.

## Abbreviations

The following abbreviations are used in this manuscript:

CBF	Cerebral blood flow
CHS	Hyperperfusion syndrome
CT	Computed tomography
DWI	Diffusion-weighted imaging
ICG	Indocyanine green
MMD	Moyamoya disease
MCA	Middle cerebral artery
MRI	Magnetic resonance imaging
MRA	Magnetic resonance angiography
PAA	Posterior auricular artery
SPECT	Single-photon emission computed tomography
STA	Superficial temporal artery
